# Challenge and hindrance academic stressors, emotional exhaustion, and GPA: a prospective three-wave study

**DOI:** 10.3389/fpsyg.2026.1984285

**Published:** 2026-10-14

**Authors:** Ke Wang, Jingyuan Li

**Affiliations:** 1 Department of Education, Liaoning Normal University, Dalian, China; 2 College of Food Industry, Shandong Institute of Commerce and Technology, Jinan, China; 3 School of Marxism, Qilu Medical University, Zibo, Shandong, China

**Keywords:** academic stress, challenge stressors, emotional exhaustion, grade point average, hindrance stressors, university students

## Abstract

**Background:**

Academic stress may have different consequences depending on whether demands are experienced as challenges or hindrances. This study examined prospective associations of challenge and hindrance academic stressors with subsequent semester grade point average (GPA) and evaluated emotional exhaustion as an intermediate variable.

**Methods:**

A multicenter, prospective three-wave observational study included 1,200 undergraduate students from four universities in Shandong Province, China. Challenge and hindrance academic stressors were assessed at baseline (T1), emotional exhaustion at T2, and end-of-semester grade point average (GPA) at T3. Multivariable regression models adjusted for baseline GPA and relevant student characteristics. Indirect associations through emotional exhaustion were estimated using 5,000 bootstrap resamples.

**Results:**

Hindrance academic stressors were associated with lower T3 GPA after full adjustment (B = −0.115, 95% CI −0.131 to −0.099), whereas challenge academic stressors showed a small positive adjusted association with subsequent GPA (B = 0.026, 95% CI 0.017 to 0.036, *p* < 0.001), despite showing essentially no bivariate association with T3 GPA (r = −0.007, *p* = 0.810). Hindrance stressors were strongly associated with subsequent emotional exhaustion (*B* = 0.587, 95% CI 0.556 to 0.618). After inclusion of exhaustion, the hindrance-stressor coefficient attenuated to −0.035, with an indirect association of −0.080 (95% bootstrap CI −0.099 to −0.062). However, hindrance stressors and emotional exhaustion showed limited empirical distinctiveness. In the sensitivity analysis restricted to institutionally verified GPA, the challenge–stressor association was attenuated and not statistically supported (*B* = 0.018, 95% CI −0.0003 to 0.037).

**Conclusion:**

Hindrance academic stressors were associated with greater subsequent emotional exhaustion and lower GPA, whereas the adjusted association between challenge stressors and GPA was small and less robust in sensitivity analysis. The indirect association through emotional exhaustion should be interpreted cautiously given the substantial empirical overlap between hindrance stressors and emotional exhaustion.

## 1 Introduction

Academic stress has become a consequential challenge in higher education because university students must simultaneously manage assessment demands, workload, competition, uncertainty about future employment, and pressures outside the classroom. Although some degree of academic demand may stimulate effort and engagement, sustained or poorly manageable stress has been associated with impaired concentration and motivation, psychological distress, sleep disturbance, reduced academic achievement, and greater risk of disengagement from education (Pascoe et al., 2020). Importantly, academic stressors should be distinguished from students’ psychological responses to those stressors. In the present study, challenge and hindrance refer to characteristics of perceived academic demands: challenge stressors are demands that may offer opportunities for mastery or achievement, whereas hindrance stressors are demands perceived as obstructive, unnecessarily constraining, or difficult to overcome (LePine et al., 2004; LePine et al., 2005). Emotional exhaustion, examined subsequently, represents a psychological response to sustained academic demands rather than an academic stressor itself. This distinction is particularly relevant in universities, where apparently similar academic demands may therefore have different consequences depending on whether students experience them as challenges or barriers.

Emotional exhaustion provides a plausible psychological pathway linking these different types of academic stressors to subsequent grade point average (GPA). Within the broader construct of student burnout, emotional exhaustion refers specifically to feelings of emotional depletion and fatigue arising from sustained academic demands; it represents one component of burnout rather than the complete burnout syndrome (Cavanaugh et al., 2000). Hindrance demands may be particularly relevant to exhaustion because they require students to expend effort on obstacles that impede goal attainment, such as unclear expectations, administrative barriers, or conflicting academic requirements. Such demands may consume psychological resources without providing a corresponding sense of progress or mastery. Challenge demands may also require considerable effort, but because they are more compatible with learning, mastery, or goal attainment, their relationship with exhaustion may be weaker or qualitatively different. Emotional exhaustion, in turn, may undermine academic performance by reducing the psychological resources available for sustained concentration, effort, and engagement with academic tasks. Accordingly, emotional exhaustion may represent an intermediate psychological response through which hindrance academic stressors are associated with poorer subsequent GPA.

Evidence across geographical and socioeconomic settings supports associations among academic stress, psychological resources, student burnout, and academic outcomes, although important inconsistencies remain. Because the present study focuses specifically on emotional exhaustion rather than the broader multidimensional construct of burnout, evidence concerning burnout is relevant primarily insofar as it demonstrates the academic consequences of its exhaustion component. Studies from the United States and Australia have demonstrated relationships among academic stress, psychological resources, and educational outcomes (Zajacova et al., 2005; Hitches et al., 2022; Baik et al., 2022), while European evidence has linked study-related burnout with poorer academic progression and achievement (Asikainen et al., 2020; Hyytinen et al., 2022). A meta-analysis encompassing more than 100,000 students similarly reported negative associations between student burnout and academic achievement, including specifically for exhaustion (Madigan and Curran, 2021). Studies from Pakistan and Nigeria have also reported relationships between academic stress and academic performance or related psychological characteristics (Javaid et al., 2024; Kamal et al., 2020; Oduwaiye et al., 2017). Within China, academic burnout is common among university students (Liu et al., 2023), while recent research indicates that stress may operate through interconnected psychological processes rather than as an isolated exposure (Chen and Chen, 2025; Ouyang et al., 2026; Dong and Luo, 2024; Ma, 2025). Collectively, this literature suggests that understanding academic outcomes requires consideration not only of the level of academic demands but also of the nature of those demands and the psychological responses associated with them. In the present study, semester grade point average (GPA) was used as the academic outcome. GPA is treated here as a specific indicator of attained academic results rather than as a comprehensive measure of the broader constructs of academic achievement, academic performance, or academic success, which are not conceptually interchangeable (Mašková and Kučera, 2022). Despite this growing literature, three limitations restrict current understanding. First, many university studies use global measures of academic stress, potentially obscuring differences between challenge and hindrance academic stressors. Second, much of the available evidence is cross-sectional, making it difficult to determine whether academic stressors precede subsequent exhaustion and academic outcomes. Third, although emotional exhaustion has been associated with student outcomes, comparatively little evidence has tested its specific role as an intermediate variable within a temporally ordered challenge–hindrance framework while controlling for prior GPA. Distinguishing emotional exhaustion from the broader burnout construct is important here because the present study examines whether this specific dimension of psychological strain helps statistically account for the prospective association between academic stressors and subsequent GPA. This distinction is important because an association between stress and later GPA may simply reflect pre-existing differences in GPA unless baseline GPA is explicitly accounted for. The present three-wave study, therefore, examined the prospective associations of challenge and hindrance academic stressors with subsequent semester GPA, with emotional exhaustion assessed at the intermediate wave, while adjusting for baseline GPA and relevant student characteristics. The study further examined whether emotional exhaustion statistically accounted for part of the association between hindrance academic stressors and subsequent GPA and whether challenge academic stressors showed a different prospective pattern.

Taken together, the challenge–hindrance framework and evidence concerning emotional exhaustion suggest a temporally ordered model in which hindrance academic demands are expected to be particularly strongly associated with subsequent emotional exhaustion, which in turn is expected to be associated with poorer subsequent GPA. Challenge academic stressors are expected to show a weaker or less adverse pattern. Based on this rationale, three hypotheses were tested. H1: Higher hindrance academic stressors will be associated with lower subsequent GPA, whereas challenge academic stressors will show a weaker or non-adverse association with GPA. H2: Hindrance academic stressors would be positively associated with subsequent emotional exhaustion, with a substantially weaker association expected for challenge academic stressors. H3: Emotional exhaustion will statistically account for part of the prospective association between hindrance academic stressors and subsequent GPA.

## 2 Materials and methods

### 2.1 Study design and setting

A multicenter, prospective three-wave observational study was conducted among undergraduate students from four universities in Shandong Province, China, between April and July 2023. The study examined prospective associations among challenge and hindrance academic stressors, emotional exhaustion, and subsequent semester GPA.

### 2.2 Study duration and measurement schedule

Data were collected at three waves. Semester GPA was used as the academic outcome. Following recommendations to distinguish specific academic indicators from broader constructs such as academic achievement, performance, and success, GPA is treated here as an indicator of attained academic results rather than as a comprehensive measure of these multidimensional constructs. Baseline assessment (T1; 10–25 April 2023) included demographic and academic characteristics, baseline grade point average (GPA), challenge and hindrance academic stressors, and sleep-related information. Emotional exhaustion, academic disengagement, and low accomplishment were assessed at T2 (5–20 June 2023). End-of-semester GPA and secondary academic outcomes were recorded at T3 (10–25 July 2023). Unique anonymized study identifiers were used to link participants across waves.

### 2.3 Study population, sampling, and eligibility

Undergraduate students were recruited from four participating universities in Shandong Province using a multicenter institution-based recruitment approach. A designated research support person at each university facilitated standardized recruitment and data collection. Students were eligible at baseline if they were enrolled at a participating university, completed the baseline assessment, and could be followed across subsequent waves using anonymized study identifiers. Of 1,850 students initially approached, 530 declined participation, leaving 1,320 participants enrolled at T1. During follow-up, 70 participants were not retained at T2, and a further 50 were not retained at T3. The final three-wave analytical cohort, therefore, comprised 1,200 participants with complete linkable data across T1, T2, and T3. Participant recruitment and follow-up are summarized in Figure 1.

**Figure 1:**
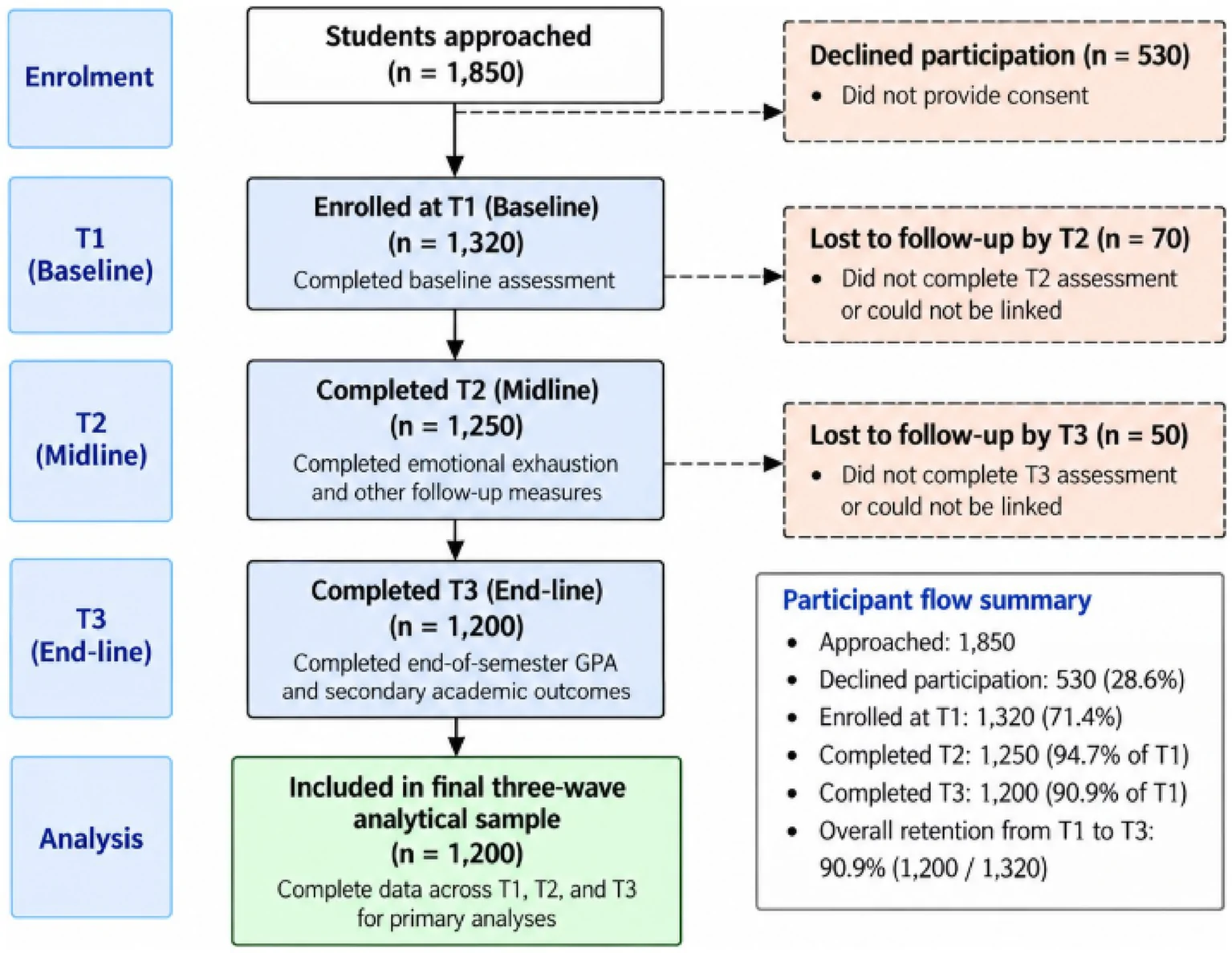
Participant recruitment, three-wave follow-up, and inclusion in the final analytical sample. T1 = baseline (10–25 April 2023); T2 = midline (5–20 June 2023); T3 = end-line (10–25 July 2023).

### 2.4 Sample-size rationale

No formal hypothesis-specific *a priori* power calculation was used to determine recruitment. The study sought a large multicenter cohort across the four participating universities while allowing for attrition across the three assessment waves. Of the 1,320 students enrolled at T1, 1,200 completed all three waves and constituted the primary analytical sample. As a sensitivity assessment, a sample of 1,200 provides 80% power at a two-sided α of 0.05 to detect a correlation of approximately *r* = 0.081, indicating sensitivity to small bivariate associations. Because the primary analyses involved multivariable adjustment and indirect associations, inference was based on effect estimates and 95% confidence intervals rather than this bivariate threshold; uncertainty around indirect associations was estimated using 5,000 bootstrap resamples.

### 2.5 Data collection and study measures

Questionnaire-based measures were administered according to the predefined three-wave schedule. The stress and burnout-related items were adapted from the cited conceptual frameworks by rewriting their content to refer to demands and experiences in the university academic setting. The study did not administer the original instruments in their complete standardized form; the adapted measures are therefore described as study-specific, context-adapted measures throughout. At T1, data were collected on age, gender, academic discipline, year of study, first-generation university status, financial strain, scholarship status, weekly study and paid-work hours, sleep quality/disturbance, baseline GPA, and challenge and hindrance academic stressors. T2 included emotional exhaustion, academic disengagement, and low accomplishment, while T3 included end-of-semester GPA, failed courses, absences, and course withdrawal.

### 2.6 Measure adaptation and pretesting

The source constructs and item content were adapted from the English-language frameworks and rendered in Chinese for administration; no formal forward–back translation procedure was undertaken. Before the main study, the adapted items underwent content review by six academic experts: two senior professors specializing in higher education administration and sociology, two research psychologists with expertise in psychometrics and student burnout measurement, and two university student counselors. The panel reviewed the items for construct relevance, clarity, and appropriateness for the Chinese university context. The resulting questionnaire was subsequently pretested with 45 undergraduate students who did not participate in the main study. The pretest assessed item comprehensibility, contextual relevance, and questionnaire flow before implementation of the three-wave study. Exact item wording and scoring are provided in Supplementary Table S1.

Challenge and hindrance academic stressors were assessed at T1 using eight items adapted from the challenge–hindrance stressor framework of Cavanaugh et al. and its application to learning settings by LePine et al. (Cavanaugh et al., 2000; LePine et al., 2004). The original framework was adapted to the university setting by rewriting item content around academic rather than occupational demands. Four items represented potentially growth-oriented demands, including workload, task complexity, time pressure, and examination rigor, while four represented obstructive demands, including administrative barriers, unclear expectations, institutional impediments, and conflicting academic requirements. Items were rated on a five-point Likert scale, and separate mean scores were calculated for challenge and hindrance stressors. Higher scores indicated greater levels of the corresponding type of academic stressors. The item wording and scoring are provided in Supplementary Table S1.

At T2, three burnout-related domains were assessed: emotional exhaustion, academic disengagement, and low accomplishment. These were treated as related aspects of student burnout rather than as interchangeable measures of burnout as a whole. Emotional exhaustion was assessed using three items adapted to the academic context from the student–burnout framework (Schaufeli et al., 2002). The items captured emotional depletion, exhaustion following sustained academic activity, and weariness associated with persistent academic demands. Three additional items assessed academic disengagement, including loss of interest, psychological distancing from academic activities, and questioning the value of one’s studies. A further three items assessed low accomplishment, including difficulty dealing with academic problems, perceived ineffectiveness, and reduced perceptions of academic contribution. All items used a five-point response scale. Because these were shortened, context-adapted measures rather than the complete original subscales, they are described as adapted burnout-related measures throughout. Exact item content and scoring are provided in Supplementary Table S1.

Psychometric screening was conducted before the prospective models were specified. Emotional exhaustion showed acceptable internal consistency (Cronbach’s *α* = 0.772) and sufficient between-participant variation for analysis. Academic disengagement showed poor internal consistency (*α* = 0.278) together with a pronounced floor distribution (mean 1.13, SD 0.24 on the 1–5 scale). Low accomplishment showed high internal consistency (*α* = 0.940), but scores were concentrated almost entirely at the lower end of the scale (mean 1.00, SD 0.08), leaving very little between-participant variation. Disengagement and low accomplishment were therefore not retained as intermediate variables in the prospective models. This decision was based on their measurement properties in the present sample rather than on their associations with academic outcomes.

Semester GPA was the primary academic outcome and was measured on a 0–4 scale. Baseline GPA at T1 represented cumulative GPA accrued before study entry, whereas T3 GPA represented GPA for the subsequent study semester only (April–July 2023) and did not incorporate academic records contributing to baseline GPA. Consistent with the conceptual distinction described in the Introduction, GPA was treated as a specific indicator of attained academic results rather than as a comprehensive measure of academic achievement, performance, or success (Mašková and Kučera, 2022). GPA source was classified as institutionally verified or self-reported, enabling a sensitivity analysis restricted to students with verified GPA at both waves. Secondary T3 outcomes included failed courses, absences, and course withdrawal.

### 2.7 Data quality, reliability, and validity

Data were screened for completeness, permissible ranges, scoring direction, and distributional abnormalities. Items designated as pre-inverted in the scoring matrix were retained in their analysis-ready direction and were not reverse-scored again. Internal consistency was evaluated using Cronbach’s alpha, together with item-level distributions and between-participant variability. Emotional exhaustion showed acceptable internal consistency (*α* = 0.772) and sufficient score variation for the planned prospective analyses. In contrast, academic disengagement showed poor internal consistency (*α* = 0.278) and a pronounced floor distribution (mean 1.13, SD 0.24 on the 1–5 scale). Low accomplishment showed high internal consistency (*α* = 0.940) but extremely limited between-participant variability (mean 1.00, SD 0.08), with scores concentrated at the lower boundary of the scale. Disengagement and low accomplishment were therefore not retained as intermediate variables in the primary prospective models. These decisions were based on their measurement properties in the present sample rather than on their associations with the study outcomes.

Given the use of context-adapted measures, their factorial structure was also examined at the item level. Confirmatory factor analysis (CFA) was performed using Mplus version 8.3 with the maximum likelihood robust (MLR) estimator to account for any potential non-normality in the item distributions. The theoretically specified three-factor structure separated challenge stressors, hindrance stressors, and emotional exhaustion, with a two-factor alternative in which hindrance-stressors and emotional-exhaustion items loaded on a common factor while challenge stressors remained separate. Model fit was evaluated using the comparative fit index (CFI), Tucker–Lewis index (TLI), root mean square error of approximation (RMSEA), and standardized root mean square residual (SRMR). Standardized factor loadings and latent-factor correlations were also examined to assess the empirical distinctiveness of the retained constructs. Because of the strong observed association between hindrance academic stressors and emotional exhaustion, an additional focused measurement analysis compared a two-factor model separating the four hindrance-stressor items from the three emotional-exhaustion items with a one-factor model in which all seven items loaded on a common factor. Discriminant validity was further examined using the latent-factor correlation and the heterotrait–monotrait ratio (HTMT).

Academic disengagement and low accomplishment were not retained as primary intermediate variables because psychometric screening indicated inadequate internal consistency/floor effects for disengagement and severely restricted between-participant variability for low accomplishment. Inter-construct correlations were examined to assess empirical overlap among the retained measures.

### 2.8 Statistical analysis

Continuous variables were summarized using means and standard deviations and categorical variables using frequencies and percentages. Pearson correlations were used to examine unadjusted associations among the principal continuous variables.

Multivariable linear regression was used to examine prospective associations of T1 challenge and hindrance academic stressors with T3 GPA, with both stressor dimensions entered simultaneously. Baseline GPA was included in all primary models. Adjusted models additionally included age, gender, academic discipline, year of study, first-generation university status, financial strain, scholarship status, weekly study hours, weekly paid-work hours, sleep quality/disturbance, and university site. T2 emotional exhaustion was subsequently added while retaining both stress dimensions to examine attenuation of the hindrance-stressor–GPA association. Model assumptions were evaluated using residual diagnostics and assessment of multicollinearity and influential observations, including variance inflation factors. An additional sensitivity analysis repeated the primary model without sleep disturbance because of its strong correlation with hindrance stressors and emotional exhaustion. Because participants were recruited from four universities, potential within-university dependence was additionally examined in a sensitivity analysis using linear mixed-effects models with a random intercept for university. The intraclass correlation coefficient (ICC) for T3 GPA was estimated from an unconditional random-intercept model, and the principal adjusted GPA models were then repeated using this multilevel specification (Supplementary Table S3).

To facilitate interpretation of effect magnitude, standardized regression coefficients (*β*) were additionally calculated for the principal continuous predictors. Incremental variance explained was evaluated using sequential models. A baseline model containing baseline GPA alone was followed by the addition of challenge and hindrance academic stressors, the prespecified covariates, and finally emotional exhaustion. Changes in *R*^2^ (ΔR^2^) were used to quantify the additional variance explained at each step.

The hypothesized indirect association of T1 hindrance stressors with T3 GPA through T2 emotional exhaustion was evaluated using regression-based pathway analysis (Hayes, 2022), with challenge and hindrance stressors entered simultaneously and adjustment for baseline GPA and the prespecified covariates. Indirect effects were estimated using 5,000 non-parametric bootstrap resamples with 95% bootstrap confidence intervals; an indirect association was considered statistically supported when the interval excluded zero. The corresponding indirect pathway for challenge stressors was estimated as a prespecified contrast. The principal GPA analysis was also repeated among participants with institutionally verified GPA at both T1 and T3. All statistical tests were two-sided, with *p* < 0.05 considered statistically significant.

### 2.9 Ethical considerations

The study protocol was reviewed and approved by the Institutional Review Board of the Shandong Institute of Commerce and Technology before commencement of the study. Participation was voluntary, and informed consent was obtained from all participants. Data were linked across waves using anonymized study identifiers, and analyses were conducted using de-identified data. The study was reported with reference to the STROBE statement (von Elm et al., 2007).

## 3 Results

Of the 1,850 students initially approached, 530 declined participation, leaving 1,320 participants enrolled at T1. During follow-up, 70 participants were lost to follow-up by T2, leaving 1,250 participants, and a further 50 were lost by T3. The final three-wave analytical sample therefore comprised 1,200 participants with complete linkable data across all three waves, corresponding to an overall retention rate of 90.9% from T1 to T3 (Figure 1). The mean age of the final analytical sample was 21.50 ± 2.28 years, and 57.1% were female. Participants were distributed relatively evenly across academic disciplines, study years, and the four participating universities. Baseline GPA was 3.09 ± 0.52, while mean challenge stressors, hindrance stressors, and T2 emotional exhaustion scores were 3.27 ± 0.99, 2.97 ± 1.14, and 1.98 ± 0.76, respectively. End-of-semester GPA at T3 was 3.20 ± 0.54. Detailed demographic, academic, psychological, and follow-up characteristics of the final analytical sample are presented in Table 1. Baseline characteristics were compared between participants retained through T3 (*n* = 1,200) and those lost to follow-up (*n* = 120). The groups were similar in baseline GPA, challenge academic stressors, age, weekly paid-work hours, and sleep disturbance (all *p* > 0.05). However, retained participants reported higher hindrance academic stressors (2.97 ± 1.14 vs. 2.70 ± 0.99, *p* = 0.006), greater financial strain (3.06 ± 1.43 vs. 2.71 ± 1.37, *p* = 0.009), and more weekly study hours (17.40 ± 7.26 vs. 14.32 ± 5.63, *p* < 0.001) than those lost to follow-up (Supplementary Table S2). These differences indicate some potential for selective attrition and were considered when interpreting the complete-case findings.

**Table 1:** Baseline characteristics and study measures of the analytical sample (N = 1,200).

Characteristic	*n* (%) or Mean ± SD
Demographic characteristics	
Age, years	21.50 ± 2.28
Gender	
Female	685 (57.1)
Male	513 (42.8)
Non-binary	2 (0.2)
Academic discipline	
Business	254 (21.2)
Humanities	233 (19.4)
Medicine/Health	250 (20.8)
STEM	213 (17.8)
Social Sciences	250 (20.8)
Year of study	
Year 1	323 (26.9)
Year 2	292 (24.3)
Year 3	294 (24.5)
Year 4	291 (24.3)
First-generation university student	370 (30.8)
Scholarship recipient	220 (18.3)
Financial strain score (1–5)	3.06 ± 1.43
Weekly study hours	17.40 ± 7.26
Weekly paid-work hours	7.93 ± 8.39
Participating university/siteᵃ	
University 1	274 (22.8)
University 2	295 (24.6)
University 3	327 (27.3)
University 4	304 (25.3)
T1 measures	
Baseline GPA (0–4)	3.09 ± 0.52
Challenge academic stressors (1–5)ᵇ	3.27 ± 0.99
Hindrance academic stressors (1–5)ᵇ	2.97 ± 1.14
Sleep quality/disturbance	3.01 ± 0.66
T1 GPA sourceᶜ	
Institutionally verified	592 (49.3)
Self-reported	608 (50.7)
T2 measure	
Emotional exhaustion (1–5)ᵈ	1.98 ± 0.76
T3 academic outcomes	
End-of-semester GPA (0–4)	3.20 ± 0.54
Courses failed, *n*	0.42 ± 0.66
Absences, *n*	7.55 ± 3.24
Course withdrawalᵉ	0 (0.0)
T3 GPA sourceᶜ	
Institutionally verified	606 (50.5)
Self-reported	594 (49.5)

Data are presented as mean ± standard deviation (SD) for continuous variables and *n* (%) for categorical variables. Percentages may not total exactly 100% because of rounding.

^a^Participating institutions are presented using anonymized identifiers (Universities 1–4) to preserve institutional confidentiality.

^b^Challenge and hindrance academic stressors were assessed at T1 using four-item composite measures; higher scores indicate higher levels of the corresponding construct.

^c^GPA source indicates whether the GPA value was obtained from an institutional record or reported by the participant.

^d^Emotional exhaustion was assessed at T2 using a three-item composite measure, with higher scores indicating greater exhaustion.

^e^No course withdrawals were recorded during the T3 follow-up period; therefore, course withdrawal was not suitable for subsequent inferential analysis.

### 3.1 Reliability and bivariate associations

Challenge academic stressors, hindrance academic stressors, and emotional exhaustion showed acceptable to high internal consistency, with Cronbach’s α values of 0.890, 0.922, and 0.772, respectively. The item-level measurement analysis indicated substantial overlap between hindrance academic stressors and emotional exhaustion. The theoretically specified three-factor model separating challenge stressors, hindrance stressors, and emotional exhaustion showed acceptable overall fit [χ^2^(41) = 196.97, CFI = 0.983, TLI = 0.978, RMSEA = 0.056, SRMR = 0.024], but the latent correlation between hindrance stressors and emotional exhaustion was 1.000. A focused analysis of the seven hindrance-stressor and emotional-exhaustion items produced a similar result. The two-factor model separating these constructs showed χ^2^(13) = 150.90, CFI = 0.979, TLI = 0.966, RMSEA = 0.094, and SRMR = 0.026, while the corresponding one-factor model showed χ^2^(14) = 150.90, CFI = 0.979, TLI = 0.969, RMSEA = 0.090, and SRMR = 0.026. The latent correlation between hindrance stressors and emotional exhaustion in the two-factor model was 1.000, and HTMT was 1.08, indicating inadequate discriminant validity between the two adapted measures. Taken together, these findings provided limited evidence that the two adapted measures were empirically distinct in this sample. Hindrance academic stressors at T1 were strongly positively associated with emotional exhaustion at T2 (*r* = 0.913, *p* < 0.001) and negatively associated with end-of-semester GPA at T3 (*r* = −0.260, *p* < 0.001). Emotional exhaustion was also negatively associated with T3 GPA (*r* = −0.265, *p* < 0.001). In contrast, challenge academic stressors showed no significant bivariate association with either emotional exhaustion (*r* = −0.024, *p* = 0.412) or T3 GPA (*r* = −0.007, *p* = 0.810). Baseline GPA was strongly positively correlated with T3 GPA (*r* = 0.926, *p* < 0.001), supporting the importance of accounting for baseline GPA in the subsequent prospective models (Table 2).

**Table 2:** Descriptive statistics, internal consistency, and correlations among the principal study variables.

Variable	Mean ± SD	Cronbach’s α	1	2	3	4	5
1. Baseline GPA (T1)	3.09 ± 0.52	—	1.000				
2. Challenge academic stressors (T1)	3.27 ± 0.99	0.890	−0.056	1.000			
3. Hindrance academic stressors (T1)	2.97 ± 1.14	0.922	−0.031	0.015	1.000		
4. Emotional exhaustion (T2)	1.98 ± 0.76	0.772	−0.022	−0.024	0.913***	1.000	
5. End-of-semester GPA (T3)	3.20 ± 0.54	—	0.926***	−0.007	−0.260***	−0.265***	1.000

GPA, grade point average; SD, standard deviation; T1, baseline assessment; T2, second-wave assessment; T3, end-of-semester follow-up.

Values are Pearson correlation coefficients (*r*). GPA, grade point average; SD, standard deviation; T1, baseline assessment; T2, second-wave assessment; T3, end-of-semester follow-up. Cronbach’s α was calculated for challenge stressors, hindrance stressors, and emotional exhaustion; it does not apply to GPA. Higher scale scores indicate higher levels of the corresponding construct. **p < 0.001.

### 3.2 Prospective associations with end-of-semester GPA

After adjustment for baseline GPA, T1 hindrance academic stressors were associated with lower T3 GPA (B = −0.110, 95% CI −0.117 to −0.102, *p* < 0.001), whereas challenge academic stressors showed a small positive adjusted coefficient (*B* = 0.026, 95% CI 0.017 to 0.035, *p* < 0.001). The latter was small in standardized terms (*β* = 0.048) and contrasted with the absence of a bivariate association between challenge stressors and T3 GPA (*r* = −0.007, *p* = 0.810). After adjustment for demographic, academic, socioeconomic, behavioral, sleep-related, and university-site covariates, the coefficients remained similar for hindrance stressors (*B* = −0.115, 95% CI −0.131 to −0.099, *p* < 0.001) and challenge stressors (*B* = 0.026, 95% CI 0.017 to 0.036, *p* < 0.001). These findings indicate that challenge stressors did not show the clear adverse prospective pattern observed for hindrance stressors; the small positive adjusted coefficient should not be interpreted as evidence that greater exposure to challenge stressors improves GPA.

When T2 emotional exhaustion was added to the fully adjusted model, greater exhaustion was associated with lower T3 GPA (*B* = −0.138, 95% CI −0.166 to −0.109, *p* < 0.001; *β* = −0.194). The hindrance-stressor coefficient was attenuated from *B* = −0.115 (*β* = −0.243) to *B* = −0.035 (*β* = −0.074), although the residual association remained statistically significant (*p* = 0.002). The challenge-stressor coefficient remained small (*B* = 0.022, 95% CI 0.013 to 0.031, *p* < 0.001; *β* = 0.040). Baseline GPA accounted for most of the explained variance in T3 GPA (*R*^2^ = 0.857). Adding challenge and hindrance academic stressors increased explained variance by 5.6 percentage points (ΔR^2^ = 0.056); addition of the prespecified covariates increased *R*^2^ by a further 0.002; and addition of emotional exhaustion increased *R*^2^ by 0.006, yielding a final *R*^2^ of 0.921. Thus, although the final model explained a large proportion of T3 GPA variance, most of this was attributable to baseline GPA (Table 3).

**Table 3:** Multivariable associations of challenge and hindrance academic stressors with end-of-semester GPA.

Predictor	Model 1 B (95% CI)	*β*	Model 2 B (95% CI)	*β*	Model 3 B (95% CI)	*β*
Baseline GPA	0.960 (0.943 to 0.978)	0.924	0.960 (0.943 to 0.978)	0.924	0.961 (0.944 to 0.978)	0.925
Challenge academic stressors	0.026 (0.017 to 0.035)	0.048	0.026 (0.017 to 0.036)	0.048	0.022 (0.013 to 0.031)	0.040
Hindrance academic stressors	−0.110 (−0.117 to −0.102)	−0.232	−0.115 (−0.131 to −0.099)	−0.243	−0.035 (−0.057 to −0.012)	−0.074
Emotional exhaustion	—	—	—	—	−0.138 (−0.166 to −0.109)	−0.194
*R* ^2^	0.913		0.915		0.921	
ΔR^2^	0.056ᵍ		0.002		0.006	

B, unstandardized regression coefficient; β, standardized regression coefficient; CI, confidence interval; GPA, grade point average.

^a^Values are unstandardized linear regression coefficients (B) with 95% confidence intervals (CI). End-of-semester GPA at T3 was the dependent variable in all models.

^b^Model 1 included baseline GPA, challenge academic stressors, and hindrance academic stressors.

^c^Model 2 additionally adjusted for age, gender, academic discipline, year of study, first-generation university status, financial strain, scholarship status, weekly study hours, weekly paid-work hours, sleep quality/disturbance, and participating university/site.

^d^Model 3 included all Model 2 variables and additionally incorporated T2 emotional exhaustion.

^e^Baseline GPA was included in all models to account for prior GPA.

^f^Regression coefficients represent adjusted associations and should not be interpreted as causal effects.

^g^A baseline-GPA-only model explained 85.7% of the variance in T3 GPA (R^2^ = 0.857); ΔR^2^ represents the increase in explained variance relative to the preceding model.

### 3.3 Indirect association through emotional exhaustion

In the fully adjusted pathway analysis, greater hindrance academic stressors at T1 were strongly associated with greater emotional exhaustion at T2 (*B* = 0.587, 95% CI 0.556 to 0.618, *p* < 0.001). In turn, greater emotional exhaustion was independently associated with lower T3 GPA after accounting for baseline GPA, both stress dimensions, and the prespecified covariates (*B* = −0.138, 95% CI −0.166 to −0.109, *p* < 0.001). The total adjusted association between hindrance stress and T3 GPA was negative (*B* = −0.115, 95% CI − 0.131 to −0.099, *p* < 0.001) and was substantially attenuated after emotional exhaustion was incorporated into the model (direct association: *B* = −0.035, 95% CI −0.057 to −0.012, *p* = 0.002).

The bootstrapped indirect association of hindrance stressors with T3 GPA through emotional exhaustion was −0.080 (95% bootstrap CI −0.099 to −0.062). Because the confidence interval excluded zero, the results supported a statistically significant indirect association through emotional exhaustion. The corresponding challenge–stress pathway was considerably smaller and operated in the opposite direction (indirect effect = 0.004, 95% bootstrap CI 0.002 to 0.007). These findings indicate different prospective association patterns for challenge and hindrance academic stressors, although interpretation of the hindrance indirect pathway requires caution given its substantial empirical overlap with emotional exhaustion (Table 4).

**Table 4:** Direct and indirect associations of challenge and hindrance academic stressors with end-of-semester GPA through emotional exhaustion.

Pathway/effect	B	SE	95% CI/Bootstrap CI	*p*
Hindrance-stress pathway				
Hindrance stressors (T1) → Emotional exhaustion (T2), *a* path	0.587	0.016	0.556 to 0.618	<0.001
Emotional exhaustion (T2) → T3 GPA, *b* path	−0.138	0.014	−0.166 to −0.109	<0.001
Hindrance stressors → T3 GPA, total association (*c*)	−0.115	0.008	−0.131 to −0.099	<0.001
Hindrance stressors → T3 GPA, direct association (*c′*)	−0.035	0.012	−0.057 to −0.012	0.002
Indirect effect: Hindrance stressors → Exhaustion → T3 GPA (*a × b*)	−0.080	0.009ᵈ	−0.099 to −0.062	—
Challenge–stress contrast				
Challenge stressors (T1) → Emotional exhaustion (T2), *a* path	−0.031	0.009	−0.049 to −0.013	<0.001
Challenge stressors → T3 GPA, total association (*c*)	0.026	0.005	0.017 to 0.036	<0.001
Challenge stressors → T3 GPA, direct association (*c′*)	0.022	0.005	0.013 to 0.031	<0.001
Indirect effect: Challenge stressors → Exhaustion → T3 GPA (*a × b*)	0.004	0.001ᵈ	0.002 to 0.007	—

B, unstandardized regression coefficient; CI, confidence interval; GPA, grade point average; SE, standard error; T1, baseline assessment; T2, second-wave assessment; T3, end-of-semester follow-up. Models were adjusted for baseline GPA, age, gender, academic discipline, year of study, first-generation university status, financial strain, scholarship status, weekly study hours, weekly paid-work hours, sleep quality/disturbance, and university site, with challenge and hindrance stressors entered simultaneously. Indirect effects (*a × b*) and 95% bootstrap CIs were estimated using 5,000 nonparametric bootstrap resamples. An indirect association was considered statistically supported when its 95% bootstrap CI excluded zero; therefore, separate *p*-values are not reported for indirect effects. ᵈIndirect effects (*a × b*) and their standard errors were estimated using 5,000 nonparametric bootstrap resamples. Statistical support for an indirect effect was determined from the 95% bootstrap confidence interval; an interval excluding zero was considered statistically significant. A separate *p*-value is therefore not reported for the bootstrapped indirect effect. 𝒛ecause this was an observational prospective three-wave study, the indirect effects represent statistical indirect associations and should not be interpreted as establishing causal mediation.

In the sensitivity analysis restricted to participants with institutionally verified GPA at both T1 and T3 (*n* = 304), hindrance academic stressors remained negatively associated with T3 GPA (*B* = −0.052, 95% CI −0.097 to −0.006, *p* = 0.026), and emotional exhaustion remained negatively associated with T3 GPA (*B* = −0.117, 95% CI −0.177 to −0.058, *p* < 0.001). In contrast, the already small challenge-stressor coefficient was attenuated and was not statistically supported in this restricted analysis (*B* = 0.018, 95% CI −0.0003 to 0.037, *p* = 0.054; Supplementary Table S4). This sensitivity analysis further supports cautious interpretation of the small positive challenge-stressor coefficient observed in the full sample. In an additional sensitivity analysis excluding sleep disturbance from the covariate set, the principal estimates were materially unchanged, including the associations of challenge stressors (*B* = 0.022), hindrance stressors (*B* = −0.027), and emotional exhaustion (*B* = −0.136) with T3 GPA and the indirect association of hindrance stressors through emotional exhaustion (*B* = −0.082). Potential clustering by university was minimal (intraclass correlation coefficient <0.001). Repeating the principal analyses using linear mixed-effects models with a random intercept for university produced estimates that were essentially unchanged from the primary analyses (Supplementary Table S3).

The adjusted prospective associations are illustrated in Figure 2. Hindrance academic stressors at T1 showed a strong positive association with emotional exhaustion at T2 (*B* = 0.587, *p* < 0.001), which was subsequently associated with lower end-of-semester GPA at T3 (*B* = −0.138, *p* < 0.001). After inclusion of emotional exhaustion, a smaller residual negative association between hindrance stressors and T3 GPA remained (*B* = −0.035, *p* < 0.01). In contrast, challenge academic stressors showed a small negative association with emotional exhaustion (*B* = −0.031, *p* < 0.001) and a small positive direct association with T3 GPA (*B* = 0.022, *p* < 0.001). Together, these pathways illustrate distinct prospective patterns for challenge and hindrance academic stressors (Figure 2).

**Figure 2:**
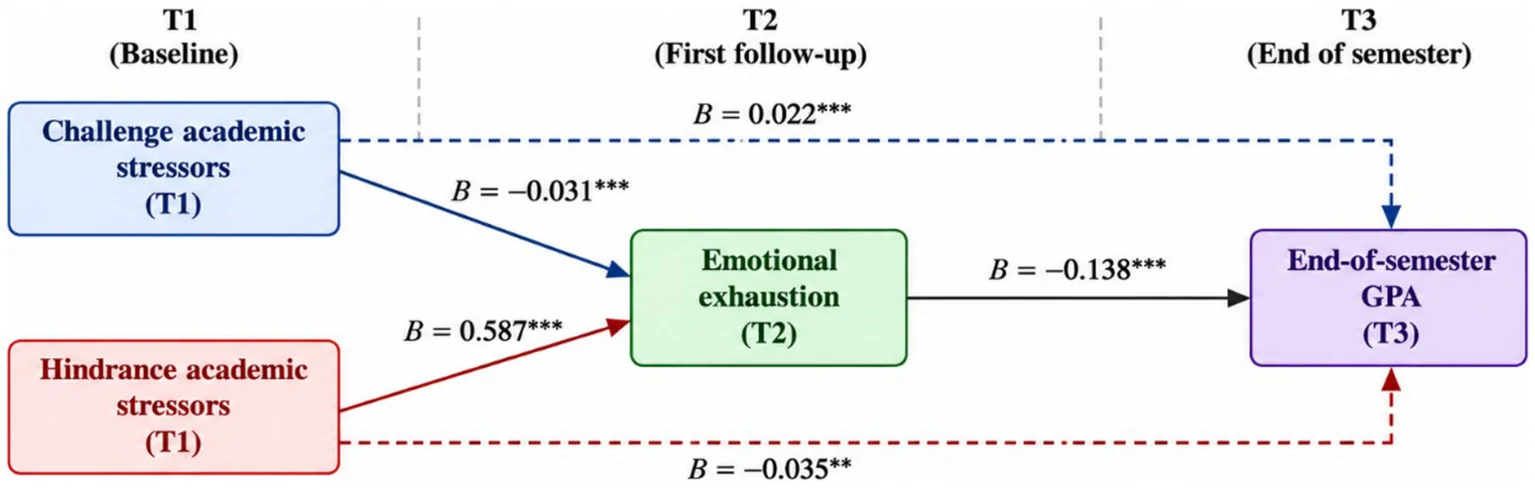
Prospective associations of challenge and hindrance academic stressors with end-of-semester GPA through emotional exhaustion. Values shown beside paths are adjusted unstandardized regression coefficients (B). Solid arrows denote paths involving T2 emotional exhaustion; dashed arrows denote direct associations with T3 GPA after inclusion of emotional exhaustion. Indirect effects were estimated using 5,000 bootstrap resamples and are reported in Table 4. **p < 0.01; ***p < 0.001.

## 4 Discussion

This three-wave study examined whether challenge and hindrance academic stressors showed different prospective associations with subsequent GPA and whether emotional exhaustion statistically accounted for part of these relationships. Three principal findings emerged. First, hindrance academic stressors at T1 were associated with poorer end-of-semester GPA at T3 after adjustment for baseline GPA and relevant student characteristics, whereas challenge academic stressors did not show the clear adverse prospective pattern observed for hindrance stressors. Second, hindrance stressors were strongly associated with greater emotional exhaustion at T2, while challenge stressors showed a small negative association with exhaustion. Third, the association between hindrance stressors and subsequent GPA was substantially attenuated after emotional exhaustion was incorporated into the model: the adjusted hindrance-stress coefficient decreased from −0.115 to −0.035, and the bootstrapped indirect association was −0.080. These findings were consistent with H1 and H2. For H3, the indirect association was statistically supported, but the lack of adequate discriminant validity between hindrance stressors and emotional exhaustion precludes interpreting this result as evidence of a clearly distinct intermediate psychological process. Nevertheless, the observational design does not establish causal effects or causal mediation.

The divergent associations of challenge and hindrance stressors with subsequent GPA are broadly consistent with the challenge–hindrance framework. Hindrance demands, including unclear expectations, administrative barriers, institutional impediments, and conflicting academic requirements, may interfere with academic goal attainment without providing corresponding opportunities for mastery. Challenge demands, in contrast, may require substantial effort while remaining compatible with academic progress. Recent evidence among Chinese university students similarly demonstrated different relationships of challenge and hindrance academic stressors with student wellbeing, supporting the relevance of distinguishing these dimensions in higher education (Zeng and Cong, 2025). Evidence from technology-related academic stress has likewise suggested that challenge and hindrance demands may show different relationships with educational outcomes (Lallmahomed, 2025). The present study extends this distinction by examining subsequent semester GPA while explicitly accounting for baseline GPA.

The small positive adjusted association between challenge stressors and T3 GPA should, however, be interpreted cautiously. Challenge stressors were essentially unrelated to T3 GPA in the unadjusted analysis (*r* = −0.007), while its adjusted coefficient was small (*B* = 0.022). Meta-analytic evidence has challenged an overly favorable interpretation of challenge stressors, showing that they can still be associated with psychological strain despite sometimes displaying more favorable relationships with performance-related outcomes than hindrance stressors (Mazzola and Disselhorst, 2019). Moreover, Webster et al. demonstrated that demands conventionally categorized as challenges or hindrances may be appraised differently across individuals and may not always fit exclusively into one category (Webster et al., 2011). Accordingly, the present findings should not be interpreted as showing that greater academic pressure improves GPA. Rather, challenge stressors exhibited a comparatively non-adverse pattern and a small positive adjusted association with subsequent GPA in this cohort.

The strong positive association between hindrance stressors at T1 and emotional exhaustion at T2, together with the much smaller association observed for challenge stressors, was consistent with H2. This pattern is consistent with research among Chinese medical postgraduates. Bao et al. found that hindrance stressors were positively associated with emotional exhaustion and that emotional exhaustion was negatively associated with academic engagement (Bao et al., 2024). Their study, however, also found that challenge stressors were positively associated with emotional exhaustion, contrasting with the small negative association observed in the present cohort. The studies differ in important respects, including the student populations and academic outcomes examined; Bao et al. studied medical postgraduates and academic engagement, whereas the present study examined undergraduates and subsequent GPA. The discrepancy therefore reinforces the need for caution in assuming that challenge demands have uniformly favorable psychological consequences. In contrast, the adverse relationship between hindrance demands and exhaustion appears more consistent.

The statistical findings provided support for the indirect association specified in H3, although the limited discriminant validity between hindrance stressors and emotional exhaustion precludes interpreting this as evidence of a clearly distinct intermediate psychological process. Greater T2 emotional exhaustion was associated with poorer T3 GPA after adjustment for baseline GPA, both stress dimensions, and the prespecified covariates. Incorporating exhaustion substantially attenuated the association between hindrance stressors and subsequent GPA, while the bootstrapped indirect association through emotional exhaustion was statistically supported. This is consistent with the broader evidence summarized earlier in the manuscript linking student burnout with poorer academic achievement (Madigan and Curran, 2021) and with findings from Bao et al. identifying emotional exhaustion as an intermediate variable in relationships between challenge–hindrance stressors and academic engagement (Bao et al., 2024). The present findings add temporal separation of stress, exhaustion, and subsequent GPA together with adjustment for baseline GPA. However, the persistence of a smaller direct association between hindrance stressors and T3 GPA indicates that emotional exhaustion did not statistically account for the entire relationship. Other mechanisms may contribute, but these were not analyzed and therefore cannot be identified from the present data.

An important feature of the results was the difference between the crude and adjusted findings for challenge academic stressors. Challenge academic stressors were essentially unrelated to subsequent GPA in the bivariate analysis (*r* = −0.007), while the positive coefficient that emerged after adjustment was small in both unstandardized and standardized terms (*B* = 0.022; *β* = 0.040 in the final model). Moreover, the coefficient was attenuated and was not statistically supported in the analysis restricted to participants with institutionally verified GPA at both waves (*B* = 0.018, 95% CI −0.0003 to 0.037, *p* = 0.054). The most defensible interpretation is therefore not that challenge stressors improve academic outcomes, but that they did not show the clear adverse prospective pattern observed for hindrance stressors in this cohort.

This interpretation is consistent with the continuing debate surrounding the challenge–hindrance framework. A recent meta-analysis of daily-diary studies found that challenge stressors had a positive direct association with performance but an adverse indirect association through strain, whereas hindrance stressors showed adverse direct and strain-mediated associations with performance (Pindek et al., 2024). These findings are particularly relevant because they illustrate that apparently challenging demands may simultaneously have favorable performance-related and unfavorable strain-related associations. Together with previous critical evaluations (Mazzola and Disselhorst, 2019; Webster et al., 2011), they argue against equating “challenge” with beneficial stress. The present findings instead suggest that distinguishing the functional characteristics of academic demands may be more informative than treating all academic stress as equivalent.

A central interpretive issue is the substantial empirical overlap between hindrance academic stressors and emotional exhaustion (*r* = 0.913). Despite their assessment at different waves and satisfactory internal consistency, the measurement analyses provided limited evidence that these constructs were empirically distinct in the present sample. Consequently, the indirect association through emotional exhaustion should not be interpreted as evidence of a clearly differentiated psychological pathway, because its magnitude may partly reflect measurement or conceptual overlap between the adapted hindrance–stressor and exhaustion measures. Future studies should evaluate more rigorously their empirical distinctiveness using fuller validated instruments, repeated measurements, and longitudinal measurement models capable of more clearly separating the constructs over time.

Several features strengthen the present study. The three-wave design temporally separated T1 challenge and hindrance stressors, T2 emotional exhaustion, and T3 end-of-semester GPA. The analytical sample comprised 1,200 students from four universities and included multiple academic disciplines and years of study. Baseline GPA was incorporated into all principal models, reducing the possibility that associations with T3 GPA simply reflected pre-existing differences in GPA. Challenge and hindrance stressors were entered simultaneously, allowing their adjusted patterns to be distinguished, and indirect effects were evaluated using 5,000 bootstrap resamples. The models also incorporated demographic, academic, socioeconomic, behavioral, sleep-related, and university-site covariates.

### 4.1 Limitations

Several limitations should be considered when interpreting these findings. First, regarding study design, the observational design precludes causal inference, and residual or unmeasured confounding remains possible despite the temporal separation of the principal measures and adjustment for baseline GPA and relevant covariates. Emotional exhaustion was also not assessed at baseline, limiting assessment of within-person changes in exhaustion over time. Second, regarding measurement, stress and emotional exhaustion were assessed using context-adapted questionnaire measures, including a shortened three-item measure of emotional exhaustion. Although the retained measures showed acceptable internal consistency and coherent item-level factor loadings, hindrance stressors and emotional exhaustion showed substantial empirical overlap. Their observed correlation was high, and the item-level factor analysis similarly provided limited evidence that the two constructs were empirically distinct. This overlap warrants particular caution when interpreting their independent coefficients and the indirect association through emotional exhaustion. GPA was not institutionally verified for all participants; however, analyses restricted to participants with institutionally verified GPA at both waves produced a broadly similar pattern of findings. Third, regarding generalizability and potential selection bias, recruitment from four universities within one Chinese province may limit generalizability to other educational settings. Although retention from T1 to T3 was high (90.9%), the primary analyses were based on participants with complete data across all three waves, and some attrition-related selection bias cannot be excluded. Although the adapted items underwent expert content review and student pretesting, no formal forward–back translation procedure was used; therefore, some linguistic or conceptual differences from the source frameworks cannot be excluded.

The findings have practical implications but should be considered within these limitations. Universities and researchers should avoid treating academic stress as a single homogeneous exposure. The clearest adverse pattern in this study involved hindrance stressors, particularly demands characterized by administrative barriers, unclear expectations, institutional impediments, and conflicting requirements. Universities may therefore consider reviewing avoidable organizational barriers, improving clarity of academic requirements, and identifying students experiencing persistent emotional exhaustion. These recommendations arise from observational associations and should not be interpreted as interventions demonstrated to be effective by this study. Conversely, the small favorable adjusted association for challenge stressors does not justify deliberately increasing student workload or academic pressure. Academic rigor and obstructive academic demands should not be considered equivalent.

Future studies should replicate these findings in geographically and institutionally diverse populations using fully validated measures and repeated assessments of stress, exhaustion, and clearly defined academic outcomes. Such designs would permit examination of reciprocal and within-person relationships and more rigorous evaluation of the empirical distinctiveness of hindrance stressors and emotional exhaustion. Prospective intervention studies would ultimately be required to determine whether reducing specific hindrance demands decreases subsequent exhaustion or improves academic outcomes.

## 5 Conclusion

Academic stress did not show a uniform prospective relationship with subsequent GPA in this three-wave cohort of Chinese university students. Hindrance academic stressors showed a relatively consistent adverse pattern, being associated with greater subsequent emotional exhaustion and lower end-of-semester GPA. Although a statistically supported indirect association through emotional exhaustion was observed, its interpretation requires caution because of the substantial empirical overlap between hindrance stressors and emotional exhaustion. In contrast, challenge academic stressors did not show an equally clear adverse pattern; their adjusted association with GPA was small and was not statistically supported in the verified-GPA sensitivity analysis. These findings support distinguishing obstructive academic demands from demanding but potentially manageable challenges rather than treating academic stress as uniformly harmful. Given the observational design and measurement limitations, further longitudinal and intervention research is needed to clarify these relationships and determine whether reducing avoidable hindrance demands can improve subsequent student outcomes.

## Data Availability

The datasets presented in this article are not readily available because: the dataset is not publicly available because it contains participant-level educational and psychological data and is subject to ethical and institutional restrictions protecting participant confidentiality. Requests to access the dataset should be directed.
